# Editorial to “Safety and feasibility of atrial fibrillation ablation after left atrial appendage closure: A single‐center experience of the left atrial appendage closure first strategy”

**DOI:** 10.1002/joa3.13098

**Published:** 2024-06-14

**Authors:** Masato Fukunaga

**Affiliations:** ^1^ Department of Cardiology Kokura Memorial Hospital Kitakyushu Japan

**Keywords:** atrial fibrillation, catheter ablation, left atrial appendage closure

Editorial comment on “Safety and feasibility of atrial fibrillation ablation after left atrial appendage closure: A single‐center experience of the left atrial appendage closure first strategy.”^1^

The management of atrial fibrillation (AF) has been sophisticated and getting more complicated because treatment options have emerged over the decades. Oral anticoagulation is still the mainstream to prevent ischemic stroke, yet sometimes difficult in patients with chronic kidney disease, elderly, and frailty. Cather ablation showed evidence to reduce heart failure hospitalization and mortality recently in a limited population, still even after the successful ablation, the recurrence of AF is casual during the longer follow‐up period. Based on their background, such as CHA_2_DS_2_‐VASc score, the continuation of oral anticoagulation is also common in daily practice.

Left atrial appendage closure (LAAC) has emerged as an alternative to long‐term anticoagulation for patients with high bleeding risk. The procedural success rate is quite high, especially using a newer generation of WATCHMAN FLX. A certain rate of patients actually need both treatment options. Recent Japanese registry data showed 32.5% of the study cohort had a history of AF ablation.^2^ A question comes up: Which comes first and how safe it is?

In the issue of Journal of Arrhythmia Chatani et al.^1^ presented new evidence to understand this clinical question. A single‐center interventional study retrospectively analyzed 46 consecutive patients with AF who had undergone CA and LAAC within 2 years. During the study period, this center performed 1992 AF ablation and 234 LAAC, which means 2.3% from the AF ablation side and 19.7% from the LAAC side. Of 46 patients, AF ablation was performed first in 31 patients and LAAC first in 15 patients. There were no differences in procedure‐related adverse events and cardiovascular adverse events after both procedures. In the AF ablation first group, four device‐related adverse events (three new peri‐device leaks and one peri‐device leak increase). They also found that three peri‐device leaks were detected with TEE at 12 months follow‐up in the early phase (within 180 days) LAAC after the AF ablation group. Events from the first procedure to the second procedure (median 7–9 months) are also interesting. More bleeding events occurred in the AF ablation first group, and a similar rate of ischemic stroke events occurred.

Combined AF ablation and LAAC is not a new idea, yet the best strategy for patients requiring both procedures needs to be elucidated. A meta‐analysis of 16 studies comprising 1428 patients showed that the pooled long‐term freedom rate from atrial arrhythmia was 0.66 (95% confidence interval [CI]: 0.59–0.71), long‐term successful rate sealing of LAAC was 1.00 (95% CI: 1.00–1.00), and ischemic stroke/transient ischemic attack/systemic embolism during follow‐up was 0.01 (95% CI: 0.00–0.02).^3^ In All but one study, AF ablation preceded LAAC, followed by 12 weeks of anticoagulation in the majority. The importance of the article by Chatani et al. is to show the trajectory of both strategies in a single‐center experience. Actually, both strategies worked equally well. More data are needed to make a tailor‐made decision on which procedure comes first.

A remained issue is the potential risk of peri‐device leak in combined procedures. It is reported that a combined procedure group had a significantly higher rate of a new residual leak than the LAAC‐alone group.^4^ The reason was explained that the resolution of ridge edema caused by radiofrequency catheter ablation might cause an increased residual leak and a smaller device compression ratio. The other group using a cryoballoon showed a similar number of residual leakage with 12 months transesophageal echocardiography follow‐up.^5^ Recently, pulse field ablation (PFA) has been rapidly introduced as a new energy source of ablation. A combined procedure of PFA and LAAC would be a way to go, and more data are coming.

Another interest for electrical physiologists is the timing of two procedures, namely, a simultaneous procedure or a sequential procedure which would be better. In Option trial [NCT03795298], a randomized control trial of either anticoagulation or LAAC in patients after AF ablation is undergoing. In the trial, either concomitant or sequential LAAC procedure is included. That would be a good help to understand this clinical question.

## FUNDING INFORMATION

N/A.

## CONFLICT OF INTEREST STATEMENT

A proctor for Boston Scientific Japan, honorarium from Boston Scientific Japan.

## ETHICS STATEMENT

N/A.

## PATIENT CONSENT STATEMENT

N/A.

## CLINICAL TRIAL REGISTRATION

N/A.

## PERMISSION TO REPRODUCE MATERIAL FROM OTHER SOURCES

None.

## Data Availability

None.
